# YTK Display-and-Secrete: Screening for Optimal Protein Secretion Elements in *Saccharomyces cerevisiae*

**DOI:** 10.1021/acssynbio.5c00264

**Published:** 2025-08-11

**Authors:** Anastasiya Kishkevich, Klaudia Ciurkot, Tom Ellis

**Affiliations:** † Imperial Centre for Engineering Biology, Imperial College London, London SW7 2AZ, U.K.; ‡ Department of Bioengineering, Imperial College London, London SW7 2AZ, U.K.

**Keywords:** yeast surface display, protein secretion, yeast toolkit, nanopore sequencing

## Abstract

Engineering yeast to secrete target proteins requires searching for optimal combinations of promoters and signal peptides so that genes can be composed that give a high expression and efficient secretion. Most methods for this involve laborious, one-by-one assessments or require the use of enzymatic reporter proteins in order to achieve high-throughput capacity. Here, we introduce a novel modular method for the high throughput screening of yeast strains designed to secrete proteins of interest. Our approach integrates combinatorial DNA assembly, yeast surface display, flow cytometry, and nanopore DNA sequencing to facilitate rapid screening. Building on a widely used yeast toolkit (YTK) for modular cloning, our system creates surface display libraries with N- and C-terminal epitope tags by fast DNA assembly and genome-integration into *Saccharomyces cerevisiae*. Flow cytometry with fluorescently labeled epitope-binding antibodies identifies strains that secrete and display the most full-length protein and can rapidly sort these from low secretors. We demonstrate our system by optimizing the secretion of the enzyme beta-lactamase and several elastin-like polypeptides (ELPs), first identifying strains with modular genetic element combinations that give the best surface display and then validating that removal of the surface-display anchor protein in these strains gives a high target protein secretion. We then show how pooled long read sequencing of sorted cells can determine the effectiveness of numerous combinations of promoters and signal peptides for a target protein in a single experiment. The data sets from this offer new insights into an optimal element choice for efficient protein secretion and could train machine learning models.

## Graphical abstract

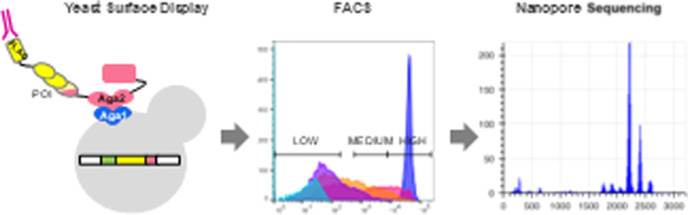

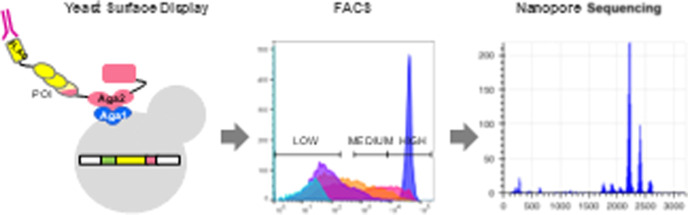

## Introduction

The industrially significant microorganism *Saccharomyces cerevisiae* offers an exceptional platform for recombinant protein production due to its well-characterized genetics, extensive manipulation tools, and protein secretion capabilities. This versatile yeast currently produces approximately 20% of biopharmaceuticals, including insulin and its analogues,^1–5^ while simultaneously gaining prominence in animal-free protein production for food applications.^6–8^ The protein expression and secretion efficiency fundamentally depend on the synergistic interaction between promoters and signal peptides, with each novel protein requiring its unique optimal combination. Comprehensive screening necessitates yeast library generation, followed by systematic evaluation.

Current methods for assessing secretion efficiency predominantly rely on enzymatic reporters for high-throughput analyses in 96-well formats,^9–14^ but when the target protein for secretion lacks enzymatic activity, this requires more laborious SDS-PAGE and Western blot methods not suitable for large numbers of samples.

Biosensor-based screening systems have emerged as sophisticated solutions to address this challenge, with several platforms developed for both *S. cerevisiae* and *Pichia. pastoris* to monitor efficient protein secretion.^15–17^ These systems employ luminescence or fluorescence reporters as quantitative readouts for secretion efficiency, offering a comprehensive approach for protein secretion analysis. However, these methods still require an individual colony isolation and sequential analysis, creating bottlenecks in high-throughput screening applications.

Yeast surface display (YSD) presents an elegant solution wherein proteins of interest fuse with anchor proteins that incorporate into the cell wall upon secretion. The pioneering YSD system, developed by Boder and Wittrup in 1997, utilized the α-agglutin mating complex (Aga1p and Aga2p).^18^ In this system, Aga2p forms disulfide bonds with Aga1p, creating display complexes incorporated into the cell wall via Aga1p. Proteins fused to Aga2p become integral components of this displayed complex with epitope tags at both termini facilitating flow cytometry detection. YSD combined with flow cytometry has since revolutionized protein and antibody engineering,^19–23^ while expanding into biocatalysis applications via Aga1p-Aga2p scaffolds for coordinated enzymatic reactions.^24–26^

The application of YSD with flow cytometry for secretion efficiency screening has been demonstrated using SAG1 as an anchor protein.^27^ This approach evaluated the human protein fragment secretion potential from a standard promoter and mating pheromone signal peptide. The Aga1p-Aga2p system has similarly proven valuable for a heterologous protein surface display and secretion screening, although these studies employed fixed promoters or signal peptides.^28–31^

Combinatorial assembly strategies successfully generate strain libraries with diverse promoters and signal peptides, an approach that has advanced heterologous protein secretion in bacteria and yeast.^14,16,17,32–36^ This combinatorial methodology, integrated with YSD, has transformed antibody engineering^21–24^ and facilitated novel signal peptide screening in *S. cerevisiae*.^25^

The Golden Gate assembly has emerged as a preferred method for yeast molecular cloning,^39,40^ enabling one-pot scarless restriction and ligation of multiple genetic components. This technique employs type IIS restriction enzymes whose recognition sites differ from cut sites, generating distinct four-base-pair overhangs despite identical restriction reactions. Strategic overhang design ensures the assembly of DNA sequences in precise configurations, facilitating modular cloning for YSD library generation.^30,37,38^

A comprehensive Golden Gate modular toolkit (yeast toolkit (YTK)) was developed for budding yeast, assigning each genetic element a specific position with unique overhangs: promoters as Part-2, coding sequences (CDS) as Part-3, and terminators as Part-4.^41^ YTK inspired a novel molecular cloning toolkit for heterologous protein production, systematically evaluating signal peptides and translation fusion proteins with various anchor proteins through flow cytometry and Western blot analysis.^30^

Our research here presents an advanced high-throughput methodology for assessing recombinant protein production in yeast. This innovative approach integrates the Golden Gate assembly, yeast surface display, FACS, and Nanopore sequencing to identify optimal promoter-signal peptide combinations for protein secretion. We have developed a novel surface display system leveraging a modular YTK-based design that enables combinatorial library generation via Golden Gate reactions. Using beta-lactamase, alpha-galactosidase Mel1, and disordered Elastin-like polypeptides as model proteins, we demonstrate the utility of our method for identifying superior secreting yeast strains.

## Results

### Design of Genetic Parts for Yeast Surface Display and Secretion

We selected Aga2p as the anchor protein for our combinatorial yeast surface display system, given its widespread use in YSD. To eliminate potential interference, we removed the endogenous *AGA1* and *AGA2* genes from the BY4741 yeast strain, preventing the formation of the wild-type α-agglutin complex. The *AGA1* gene was reintroduced into the genome using the strong constitutive promoter pCCW12, integrated at the *URA3* locus to ensure sufficient Aga1p levels for the recombinant protein display (*AGA1* ^*+*^ strain, yAK003). In our YSD design, we strategically positioned Aga2p at the C-terminus of the protein, enabling the exploration of diverse signal peptides at the N-terminus (Figure 1A).

**Figure 1:**
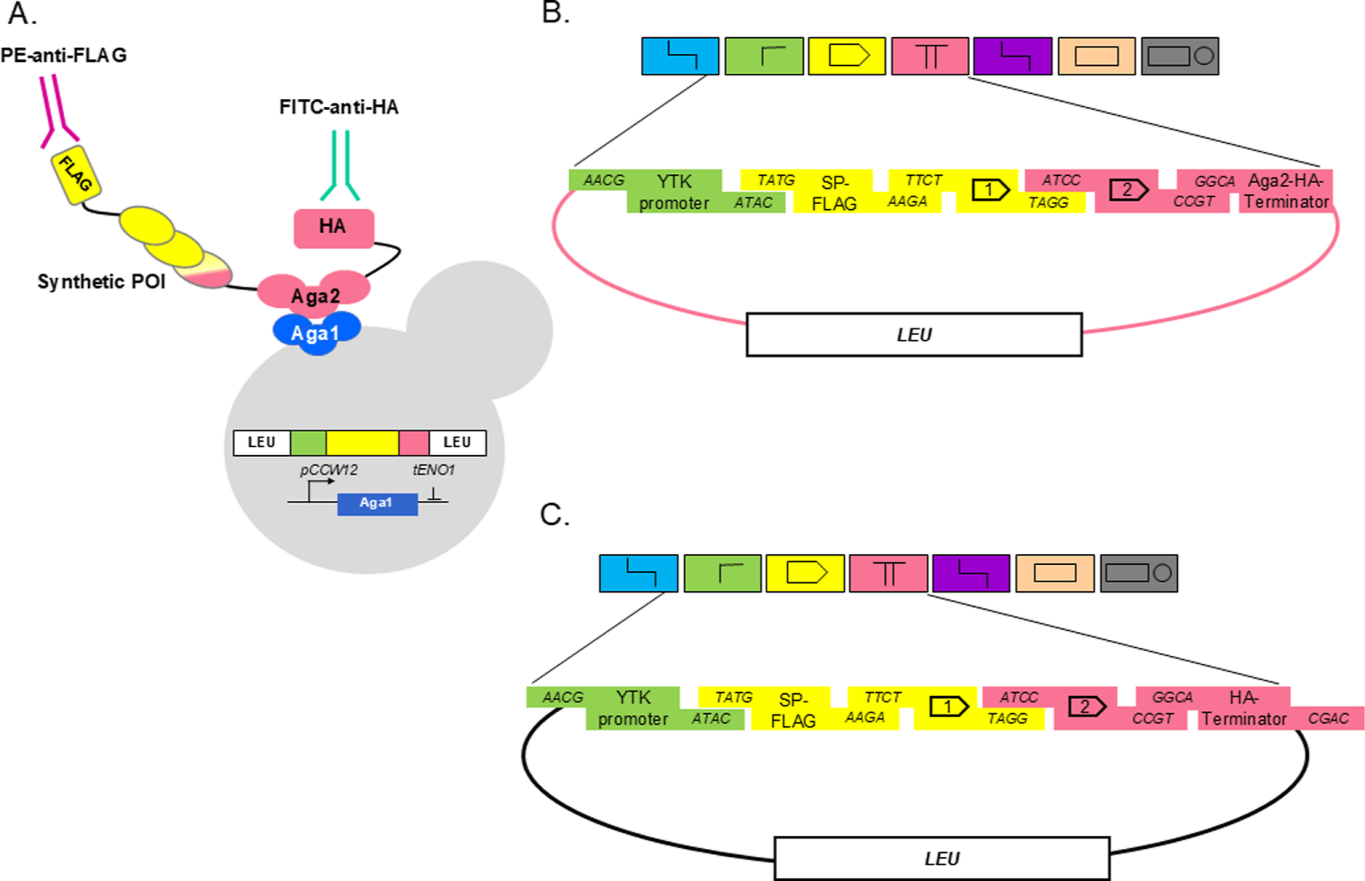
Illustration of yeast surface display and genetic designs. (A) Diagram of a resulting yeast cell with an integrated genetic construct into a LEU2 locus. A synthetic protein is displayed via Aga2 anchor protein and carries FLAG tag at the N terminus and HA tag at the C terminus. Epitope tags can be detected with fluorescence-conjugated antibodies PE-DAZZLE (anti-FLAG) and FITC-anti-HA. (B,C) Design of genetic parts and the integration vector for yeast surface display (B) and secretion (C). Promoters are from the original MoClo YTK, SP-FLAG, and CDS #1 designed as YTK parts 3a and 3b, CDS #2, and HA-terminator with or without anchor Aga2 designed as modified 4a and 4b parts.

Our genetic part design drew inspiration from the original MoClo YTK system^41^ (Figure 1B,C). Leveraging the yeast toolkit’s diverse promoter repertoire, we utilized the available YTK Part-2 promoters. The signal peptide panel comprised three native *S. cerevisiae* sequences: mating pheromone MFA1, glucoamylase STA1, and invertase SUC2, complemented by an *Aspergillus*spp alpha-amylase signal peptide. To streamline Golden Gate assembly, these signal peptides were fused with a 3xFLAG tag (3xDYKDDDDK) to facilitate surface display detection. The fused constructs were cloned into pYTK001 entry vectors as Part-3a for the subsequent combinatorial integration plasmid assembly.

We intentionally selected complex synthetic proteins to challenge our display and secretion system. Elastin-like polypeptides (ELPs), intrinsically disordered proteins composed of Valine-Proline-Glycine-X-Glycine repeats,^42^ formed the core of our experimental design. We designed a set of 5 ELP repeats (2, 4, 6, 8, and 10), with one ELP unit (ELP1) spanning 100 amino acids, structured as (VPGVG)_5_-(VPGAG)_2_-(VPGGG)_3_-(VPGVG)_5_-(VPGAG)_2_-(VPGGG)_3_. Building upon previous successful ELP secretion demonstrated in cellulose-producing bacteria and *K. phaffii*,^43^ we also incorporated a *S. cerevisiae* codon-optimized beta-lactamase (BLA) and alpha-galactosidase, previously shown to be secreted using the mating factor signal peptide.^44^ The experimental design further incorporated the cellulose-binding module from *Cellulomonas fimi* exoglucanase (CBMcex), a protein with established secretion capabilities in yeast.^41^ We modified this module to preserve a (GS)_8_ linker. The complete surface display construct included a GS linker, Aga2p coding sequence (without a signal peptide) fused to an HA tag, and the *TDH1* terminator from *S. cerevisiae*, cloned into a LEU2 integration plasmid. The resulting destination integration plasmid, pAK009, contained a modified Part-4b comprising (GS)_8_-Aga2-HA-tTDH1, LEU2 homology arms, a LEU2 selection marker (Figure 1B), and *E. coli* GFP flanked by BsaI restriction sites to facilitate the Golden Gate library assembly. All components were carefully designed with overhangs that enable an efficient Golden Gate assembly in the part order 2–3a-3b-4a into the pAK009 integration vector, generating a single open reading frame with the Aga2p and HA tag. Notably, we positioned the HA tag behind Aga2 to integrate Aga2p into the synthetic protein rather than merely as a surface display unit. To comprehensively assess protein secretion, we engineered an additional modified Part-4b lacking the anchor protein, consisting of (GS)_8_-HA-tTDH1 (Figure 1C), enabling the rapid construction of strains for the direct evaluation of protein secretion into the media.

### Generation of Surface Display Libraries via Combinatorial Assembly

First, we generated a set of strains displaying ELPs of different lengths as an initial test of the idea. The plasmids for expressing ELPs were assembled using a standard Golden Gate assembly, transforming them into chemically competent yeast cells. Plasmids consisted of a strong CCW12 promoter, the Sc alpha mating pheromone signal peptide, and a coding sequence for ELP proteins fused to a C-terminal cellulose binding motif (CBM). These plasmids were linearized in vitro and transformed into the *AGA1* ^*+*^ yeast strain to site-specifically integrate into the genome. Integrated strains were stained with fluorophore-conjugated antibodies targeting FLAG and HA tags and analyzed by flow cytometry. Single cells from all six strains demonstrated an increased signal from both PE-DAZZLE (anti-FLAG) and FITC (anti-HA) antibodies in comparison to a strain without a surface-displayed protein (Figure S1). The strain carrying ELP1 showed the highest median level for both PE-DAZZLE and FITC signals and thus was chosen as a positive control for subsequent experiments.

We then generated libraries of ELP-CBM and β-lactamase-CBM plasmids. After harvesting all positive nonfluorescent colonies and preparing the plasmid library, we transformed it into the *AGA1* ^*+*^ yeast strain (yAK03). The yeast cell pools from SD-LEU plates were recovered in rich media for three hours before flow cytometry analysis.

Our initial projections anticipated 80 unique promoter-signal peptide combinations, each potentially yielding distinct surface display levels. The libraries were treated the same way as single strains: stained with fluorophore-conjugated antibodies targeting FLAG and HA tags and subsequently analyzed by flow cytometry (Figure 2A). The results revealed remarkable fluorescence diversity within the libraries, indicating varied surface display levels across cells. Despite using identical antibody concentrations, the fluorescence profiles for HA and FLAG epitope tags differed significantly. The PE median fluorescence intensity ranged from 1 × 10^2^ (negative control) to 1 × 10^5^ (positive), while FITC ranged from 1 × 10^3^ to 1 × 10^4^. This variation likely stems from antibody-specific characteristics, resulting in a broader fluorescence intensity spread for anti-FLAG staining. Consequently, we opted to use only PE-DAZZLE staining in subsequent experiments to enable more nuanced strain isolation.

**Figure 2:**
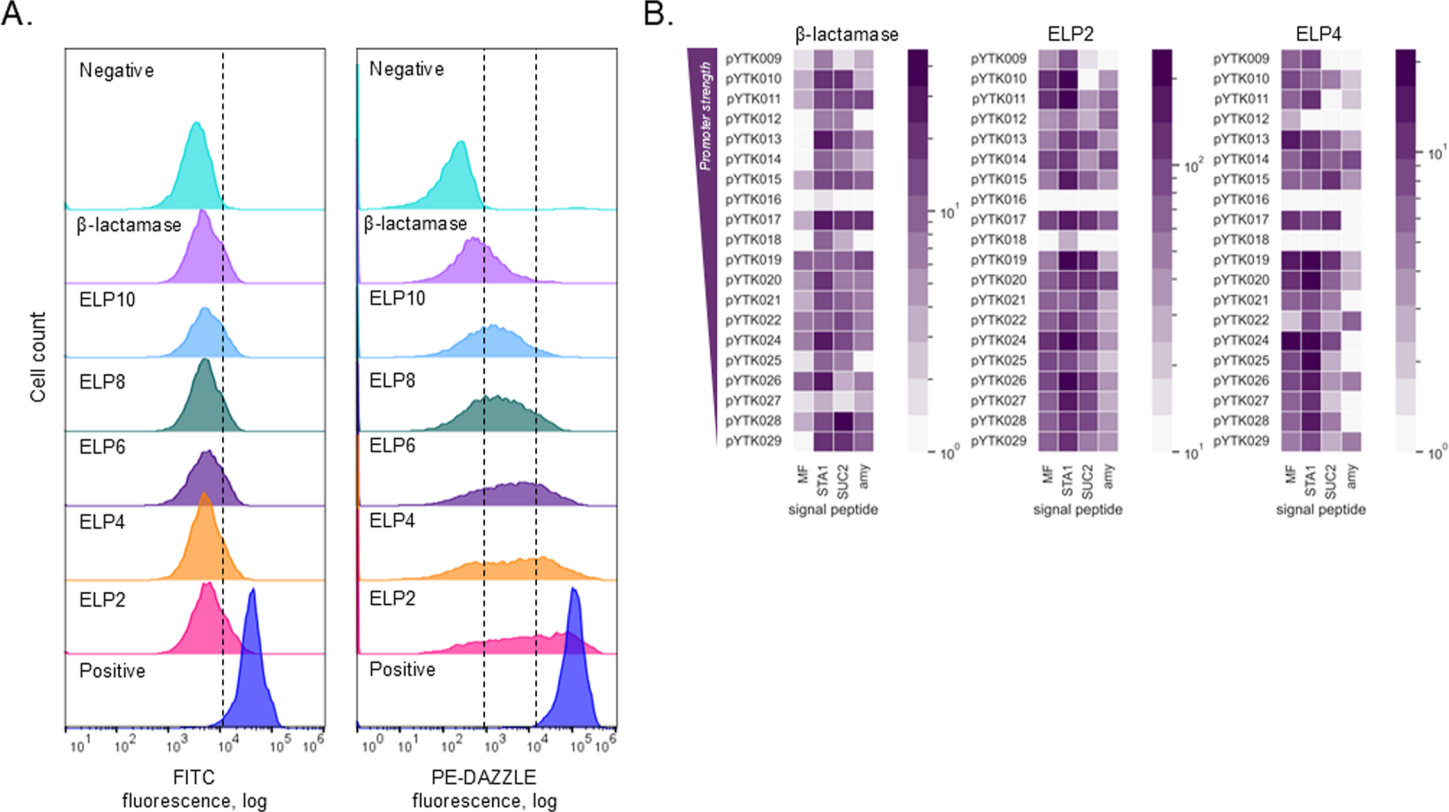
Analysis of yeast libraries displaying β-lactamase, ELP2, ELP4, ELP6, ELP8, and ELP10. (A). Flow cytometry analysis of yeast libraries. ≥ 2000 yeast colonies were collected. Cells were treated with FITC-anti-HA and PE-DAZZLE antibodies and analyzed by Attune NxT Flow Cytometer. The negative control is stained AGA1^+^ strain (without surface display), the positive control is pCCW12 MF-3xFLAG-ELP1-CBM-AGA2-HA stained. At least 10,000 events were analyzed for each sample. A dashed line separates negative and positive populations. (B) Results of nanopore sequencing of pooled amplicons from β-lactamase, ELP2, and ELP4 libraries. Heatmaps were built with the data obtained by nanopore sequencing. ≥ 1000 full reads for each library were analyzed. Each square represents a single genotype, and the intensity of the color correlates with the number of reads for each genotype within the analyzed full reads.

After preliminary screening, we selected ELP2, ELP4, and BLA libraries for detailed analysis based on their distinct fluorescence profiles. The ELP2 library exhibited a higher cell count with elevated surface display levels; ELP4 showed a more balanced distribution between negative and positive controls, while the beta-lactamase library predominantly displayed fluorescence levels comparable to the negative control.

To verify the genetic diversity underlying these fluorescence variations, we performed nanopore sequencing. Using Oxford Nanopore Technology’s capability to sequence DNA libraries with barcoded amplicons,^45^ we extracted total genomic DNA from each yeast library and amplified integrated genetic constructs using protein-specific barcodes. The sequencing results confirmed a diverse genotypic landscape within the libraries, validating that the observed fluorescence and surface display variations resulted from the successful plasmid library generation and genome integration (Figure 2B).

## Sorting Yeast Libraries and Identification of Genotypes

Our subsequent experimental phase focused on sorting yeast libraries based on the fluorescence intensity to identify promoter and signal peptide combinations, yielding diverse surface display levels (Figure 3A). The PE-DAZZLE fluorescence spectrum enabled cell segregation into low-, medium-, and high-surface-display populations (Figure 3B).

**Figure 3:**
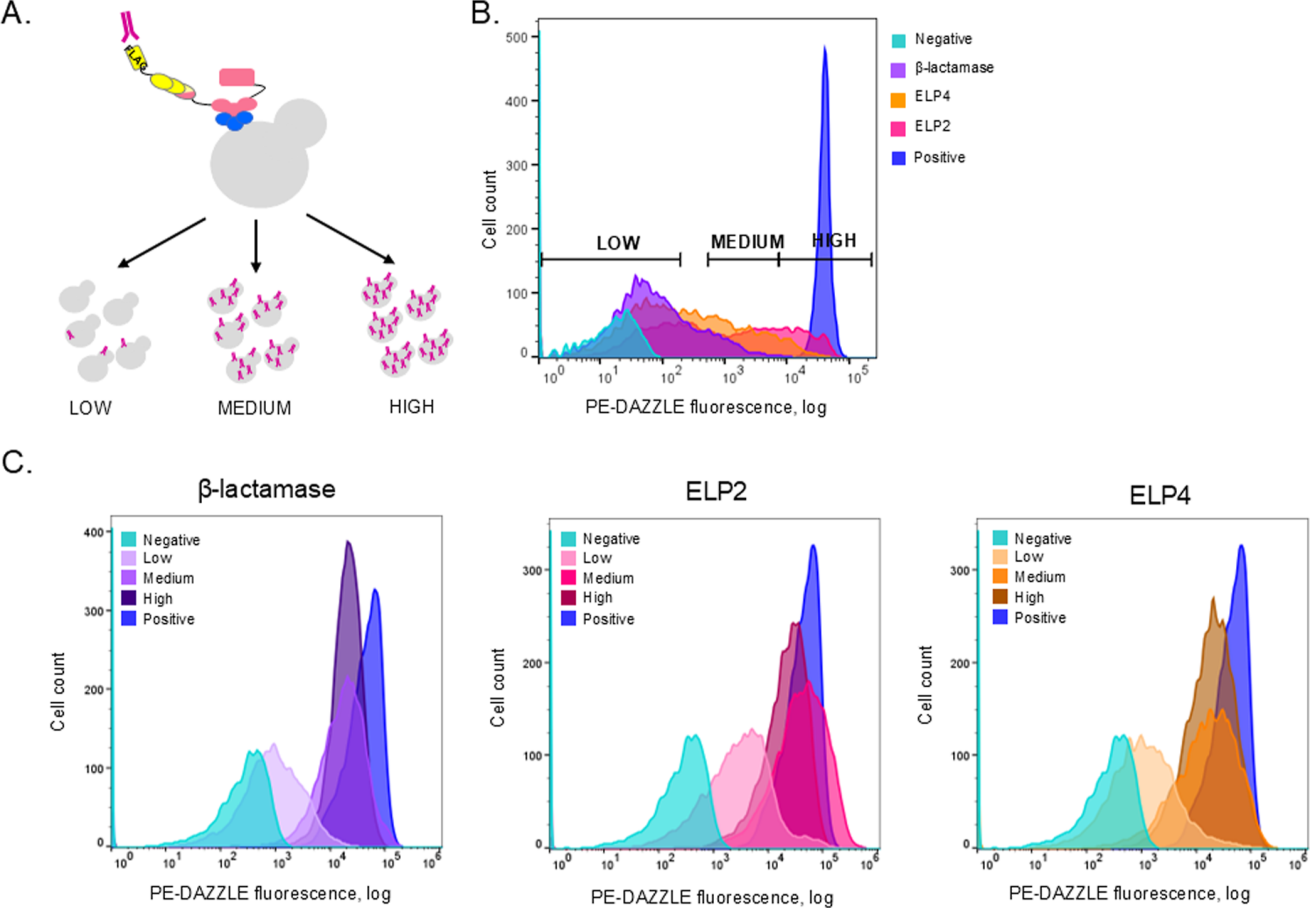
Sorting of yeast libraries. (A) Illustration of the sorting strategy into three populations based on the PE-DAZZLE fluorescence intensity. (B) Gating strategy for FACS. The sorting was performed with BD FACSAria III. 10^6^ cells were sorted into three populations based on the PE-DAZZLE fluorescence intensity. All the data for the three libraries is plotted as a histogram. Control is pYTK010 MF-3xFLAG-ELP1-CBM without staining (negative control) and with staining (positive control). (C) Flow cytometry analysis of sorted populations after overnight recovery. The recovered cells were stained with the PE-DAZZLE antibody and were analyzed by an Attune NxT Flow Cytometer. At least 10,000 events were analyzed for each sample. Control is pYTK010 MF-3xFLAG-ELP1-CBM without staining (negative control) and with staining (positive control).

During FACS analysis, we strategically excluded a subset of cells between low and medium gates to mitigate the gene expression variation and obtain more precise low population results. The high gate encompassed cells displaying surface display levels equivalent to or exceeding those of the positive control. We anticipated a potential genotype overlap between the medium and high populations. Following sorting, each cell population was cultured overnight in a rich medium and preserved as glycerol stocks.

Postrecovery, cells were stained with PE-DAZZLE antibodies and reanalyzed by flow cytometry (Figure 3C). The analysis revealed a distinct peak for the low population, while medium and high population fluorescence intensities overlapped as predicted. This overlap suggested potential genotype similarities. Note that postsorting flow cytometry was performed on a different machine, explaining the nonmirrored fluorescence profiles.

Nanopore sequencing comprehensively characterized the promoter and signal peptide combinations within each population (Figure 4). Heatmaps summarized the abundance of each combination in the sorted populations with the five most abundant combinations listed in Table 1.

**Figure 4:**
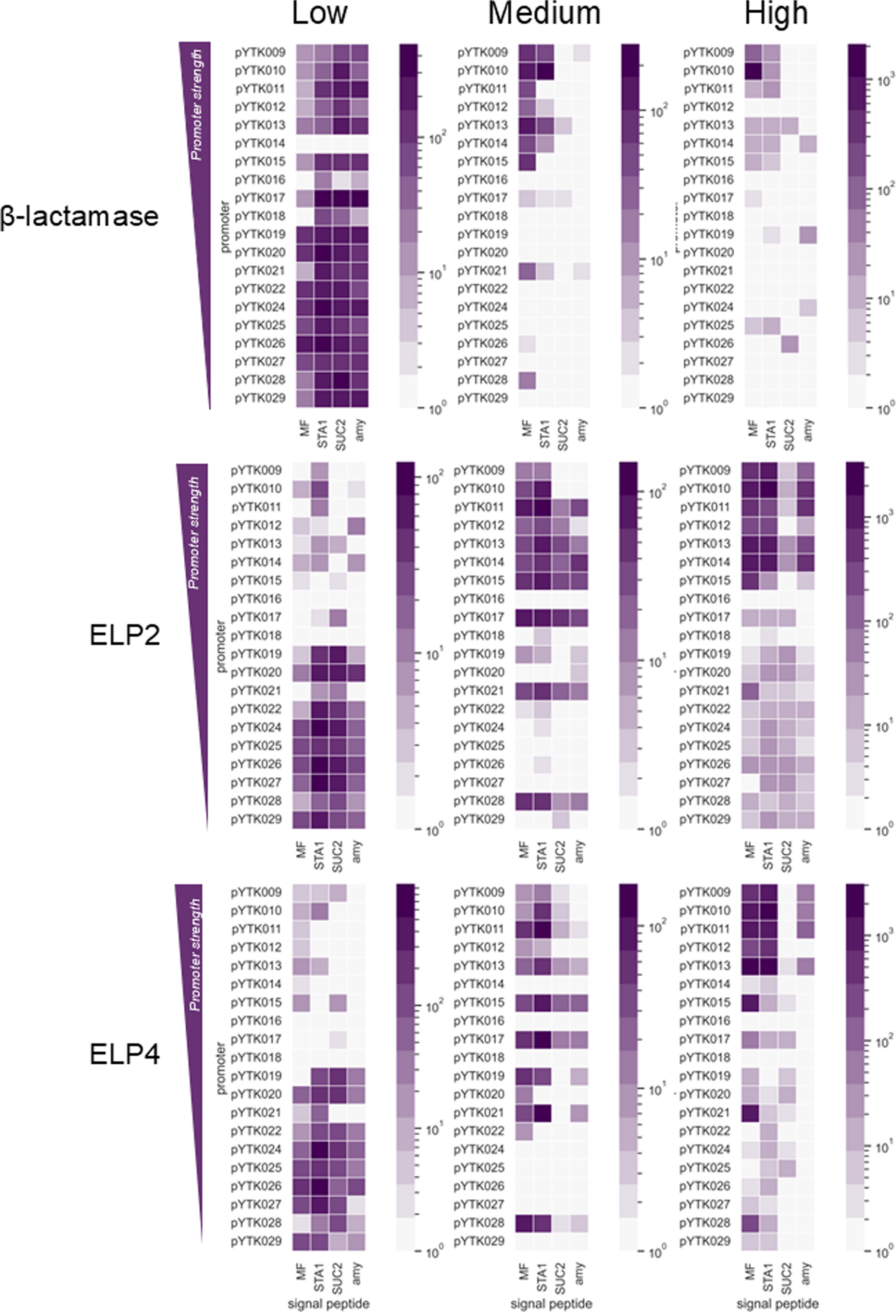
Heatmaps of different promoter and signal peptide combinations present in each sorted population. Amplicons with barcodes specific to beta-lactamase, ELP2 and ELP4, and each sorted subpopulation were sequenced with nanopore sequencing. Heatmap intensities corelate with the number of reads for each promoter and signal peptide combination identified by sequencing.

**Table 1:** Most Abundant Promoter and Signal Peptide Combinations Identified by Nanopore Sequencing in Each Population for beta-lactamase, ELP2, and ELP4

β-lactamase low		β-lactamase medium		β-lactamase high	
pYTK028_SUC2	4.9%	pYTK010_STA1	27.7%	pYTK010_MF	86.8%
pYTK026_STA1	3.5%	pYTK010_MF	16.0%	pYTK009_MF	2.8%
pYTK024_STA1	3.4%	pYTK013_MF	11.1%		
pYTK017_STA1	3.2%	pYTK015_MF	9.2%		
pYTK019_SUC2	2.6%	pYTK009_MF	6.7%		

ELP4 low		ELP4 medium		ELP4 high	
pYTK026_STA1	8.3%	pYTK011_STA1	10.3%	pYTK010_STA1	21.2%
pYTK024_STA1	7.9%	pYTK015_STA1	6.4%	pYTK009_STA1	9.0%
pYTK027_STA1	5.7%	pYTK017_MF	5.7%	pYTK014_MF	8.5%
pYTK022_STA1	4.7%	pYTK013_STA1	5.1%	pYTK014_STA1	8.1%
pYTK020_SUC2	4.6%	pYTK011_MF	4.9%	pYTK013_STA1	7.8%

ELP4 low		ELP4 medium		ELP4 high	
pYTK024_STA1	17.7%	pYTK017_STA1	13.8%	pYTK013_STA1	20.0%
pYTK026_STA1	16.8%	pYTK011_STA1	12.3%	pYTK013_MF	12.3%
pYTK027_STA1	6.2%	pYTK021_STA1	10.5%	pYTK010_STA1	10.9%
pYTK019_SUC2	5.0%	pYTK015_STA1	6.6%	pYTK015_MF	9.4%
pYTK026_MF	5.0%	pYTK028_MF	5.8%	pYTK011_MF	9.1%

For the structured enzyme beta-lactamase (BLA), almost all genotypes congregated in the low population. The top five combinations featured weak promoters (pYTK017, pYTK026, pYTK024, pYTK028, and pYTK019) and exclusively two signal peptides: glucoamylase STA1 and invertase SUC2 (Table 1).

Medium and high populations shared identical genotypes: strong promoters (pYTK009-pYTK015) and mating pheromone MF and STA1 signal peptides. Notably, one genotype dominated the high population, comprising 86.8% of reads: the *CCW12* promoter (pYTK010) coupled to the MF signal peptide.

The elastin-like polypeptides (ELPs) exhibited a distinct distribution pattern. Low populations for both ELP2 and ELP4 predominantly consisted of weak promoters yet incorporated all signal peptides. Medium populations featured strong promoters (pYTK009-pYTK015) and all four signal peptides. In the ELP2 high population, most promoters and signal peptides were identified with strong promoter combinations showing a greater abundance. In the ELP4 high population combinations of strong promoters and MF and STA1 signal peptides dominated, while the *amy* signal peptide was present exclusively in combination with four strong promoters.

An intriguing observation emerged: medium-strength promoter pYTK021 and weak-strength promoter pYTK028 appeared in both medium and high populations. However, investigating these nuanced findings falls beyond the current study’s scope.

### Validation of Nanopore Sequencing Results

Nanopore sequencing comprehensively identified all genotypes present in low, medium, and high populations across the beta-lactamase, ELP2, and ELP4 libraries. For each protein and population, we selected the five most abundant reads for validation and further investigation (Table 1).

In the beta-lactamase analysis, two genotypes from the high population were also present in the medium population, with a total of 10 genotypes being validated. We systematically assembled the identified promoter-signal peptide combinations into the surface display vector and transformed them into the *AGA1* ^*+*^ strain. Flow cytometry analysis using PE-DAZZLE antibodies (Figure 5) corroborated the sequencing results. Fluorescence intensity patterns aligned precisely with our initial observations: low population strains exhibited median fluorescence levels near the negative control, high population strains matched the positive control, and medium population strains displayed fluorescence levels intermediate between low and high. To assess beta-lactamase secretion, we constructed promoter-signal peptide combinations without surface display elements in an LEU integration vector, integrating these genetic constructs into the BY4741 wild-type strain. An enzymatic assay using nitrocefin confirmed functional enzyme secretion in the media. Critically, the secretion levels mirrored the fluorescence intensity trends: strains with a low fluorescence demonstrated correspondingly low protein secretion, while high fluorescence strains exhibited elevated secretion levels (Figure 5B). Supernatants from the low population showed minimal enzymatic activity, whereas supernatants from the medium and high population strains produced around 0.7–0.72 μg/mL of enzymes, as calculated from a standard curve of beta-lactamase activity (Figure S2).

**Figure 5:**
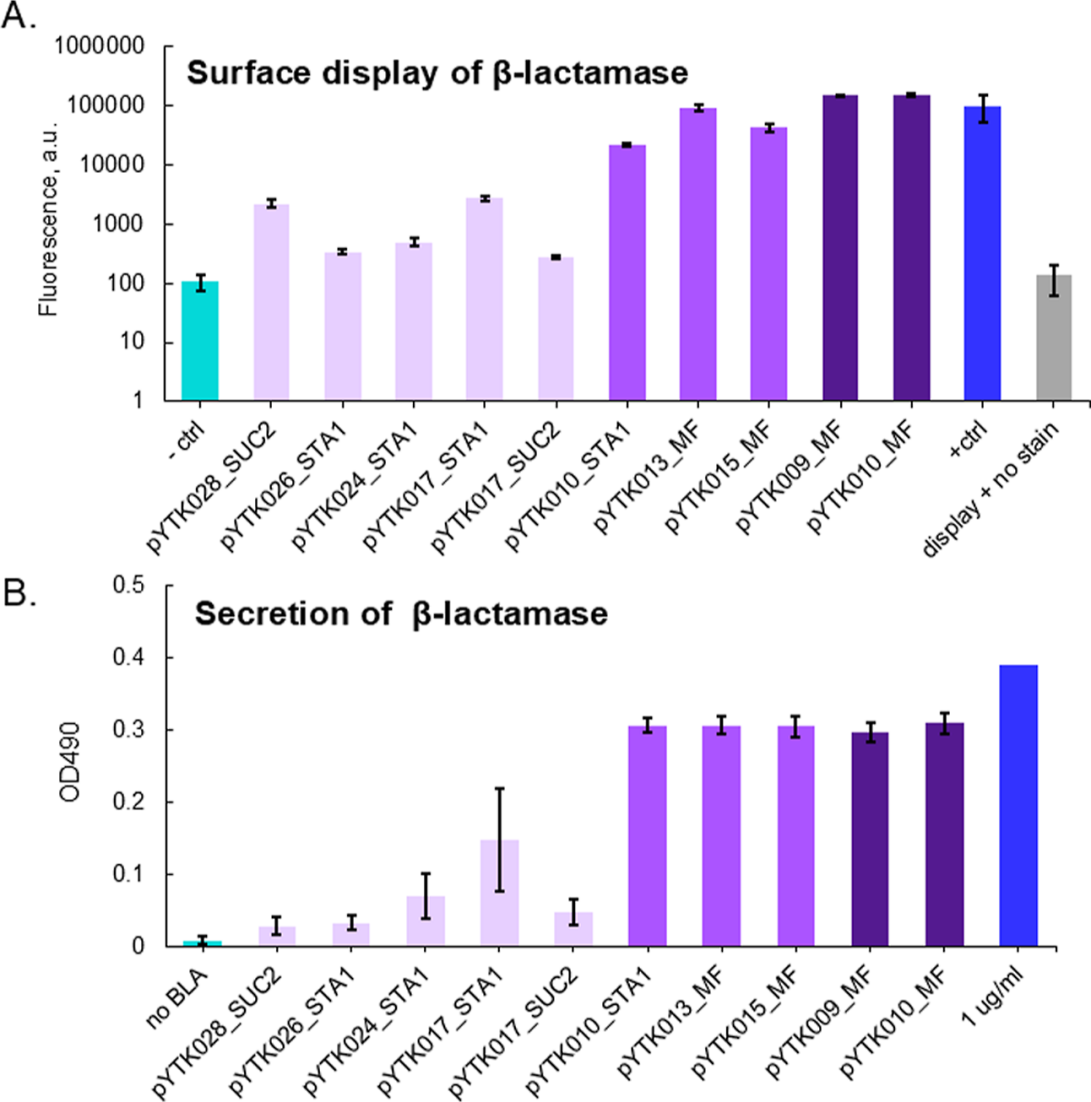
Validation of the top hits from beta-lactamase populations. The most abundant promoter and signal peptide were assembled into surface display and secretion vectors and integrated into yAK03 (A) and BY4741 (B), respectively. Pale purple—low, purple—medium, and dark purple—high population. (A) Yeast cells were stained with PE-Dazzle antibodies and fluorescence intensity was measured by flow cytometry. Three independent isolates were analyzed, data is presented as an average of median fluorescence values, *n* = 3, error bars = standard deviation. Controls are *pYTK010 MF-3xFLAG-ELP1-CBM* without staining (display + no stain) and with staining (positive control); yeast without surface display (negative control). (B) Beta-lactamase-CBM enzymatic activity assay was performed with a supernatant with 50 μg/mL of nitrocefin solution. Three independent isolates were analyzed, data is presented as an average of OD490 reads, error bars = standard deviation. Negative control is a BY4741 strain, reference is 1 μg/mL of beta-lactamase solution. Values for the supernatant are normalized to rich media optical density.

Similar validation procedures were applied to the ELP2 and ELP4 libraries. Reassembled promoter-signal peptide combinations demonstrated the anticipated fluorescence profiles: low population strains approximated the no surface display negative control, while medium and high populations closely resembled the positive control (Figure 6A,C).

**Figure 6:**
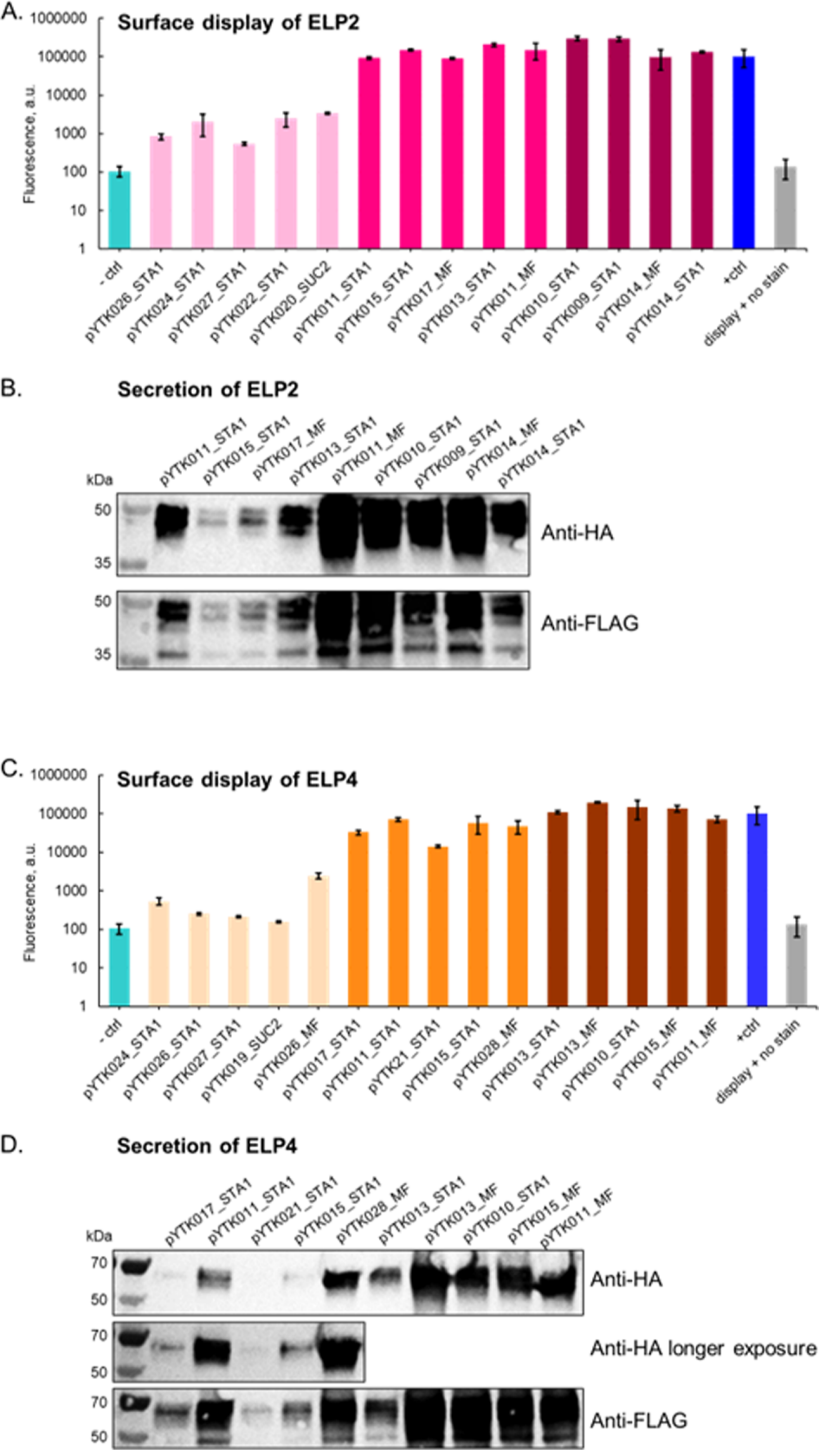
Validation of the top hits from ELP populations. The most abundant promoter and signal peptide combinations were assembled into surface display and secretion vectors and integrated into yAK03 (A,C) and BY4741 (B,D), respectively. Pale pink and orange—low, pink and orange—medium, and dark pink and brown—high population. (A,C) Yeast cells were stained with PE-Dazzle antibodies and fluorescence intensity was measured by flow cytometry. Three independent isolates were analyzed, data is presented as an average of median fluorescence values, *n* = 3, error bars = standard deviation. Controls are pYTK010 MF-3xFLAG-ELP1-CBM without staining (display + no stain) and with staining (positive control); yeast without surface display (negative control). (B,D) Levels of ELP-CBM secretion in strains from medium and high populations were assessed by the Western blot. The supernatant of overnight cultures was concentrated and loaded onto two different SDS-PAGE gels. After the transfer each membrane was probed with anti-HA and anti-FLAG primary antibodies for both N and C termini of the secreted protein. A representative Western blot is shown here.

Protein secretion was evaluated via SDS-PAGE and Western blot using 20-fold concentrated supernatants and antibodies targeting both FLAG and HA epitope tags. The Western blot revealed protein bands of consistent intensity and size for both epitope tags, suggesting specific ELP2 and ELP4 detection. Due to the potential glycosylation of the CBM fusion partner, the observed band sizes slightly exceeded expectations (Figure 6B,D). Based on a visual comparison to the reference protein, the concentration of different secreted ELP2-CBM and ELP4-CBM combinations varies from 0.5 to 2 μg/mL (Figure S3).

### Using Surface Display and Secretion Method for Single Colony Analysis

Our method presented an additional powerful application: identifying the most effective strain for protein culturing and production. While still requiring a combinatorial assembly, this approach enables downstream analysis through routine flow cytometry and Sanger sequencing. To validate this single colony methodology, we selected yeast alpha-galactosidase Mel1, a protein naturally secreted into the media, and designed its coding sequence (CDS) without a signal peptide as part 3b.

We conducted combinatorial assembly by mixing YTK promoters, signal peptides, Mel1 CDS, and CBM CDS, subsequently transforming the plasmid library into the *AGA1* ^*+*^ strain. From the transformation, we randomly selected and grew 90 single colonies to saturation, stained them with the PE-DAZZLE antibody, and performed flow cytometry analysis (Figure 7A). Based on the fluorescence intensity, 10 colonies were earmarked for further investigation. Intriguingly, wells E5 and F2 displayed the highest fluorescence levels, approximately 3.3 and 4.4 times the positive control, respectively. However, subsequent sequencing revealed that Mel1 CDS was absent in these wells. This observation suggests a cautionary note: in future experiments, strains exhibiting fluorescence levels more than three times the positive control should be approached with skepticism. The remaining colonies demonstrated varied surface display characteristics (Figure 7B). Wells H4, E8, and C6 exhibited medium surface display levels, while wells H7, H1, and A9 showed high levels of display. A notable pattern emerged: colonies in wells H1, B5, and D3 shared an identical promoter and signal peptide combination of pYTK011_SUC2. Mirroring our high-throughput approach, we validated whether these specific promoter-signal peptide combinations could facilitate full-length functional enzyme secretion.

**Figure 7:**
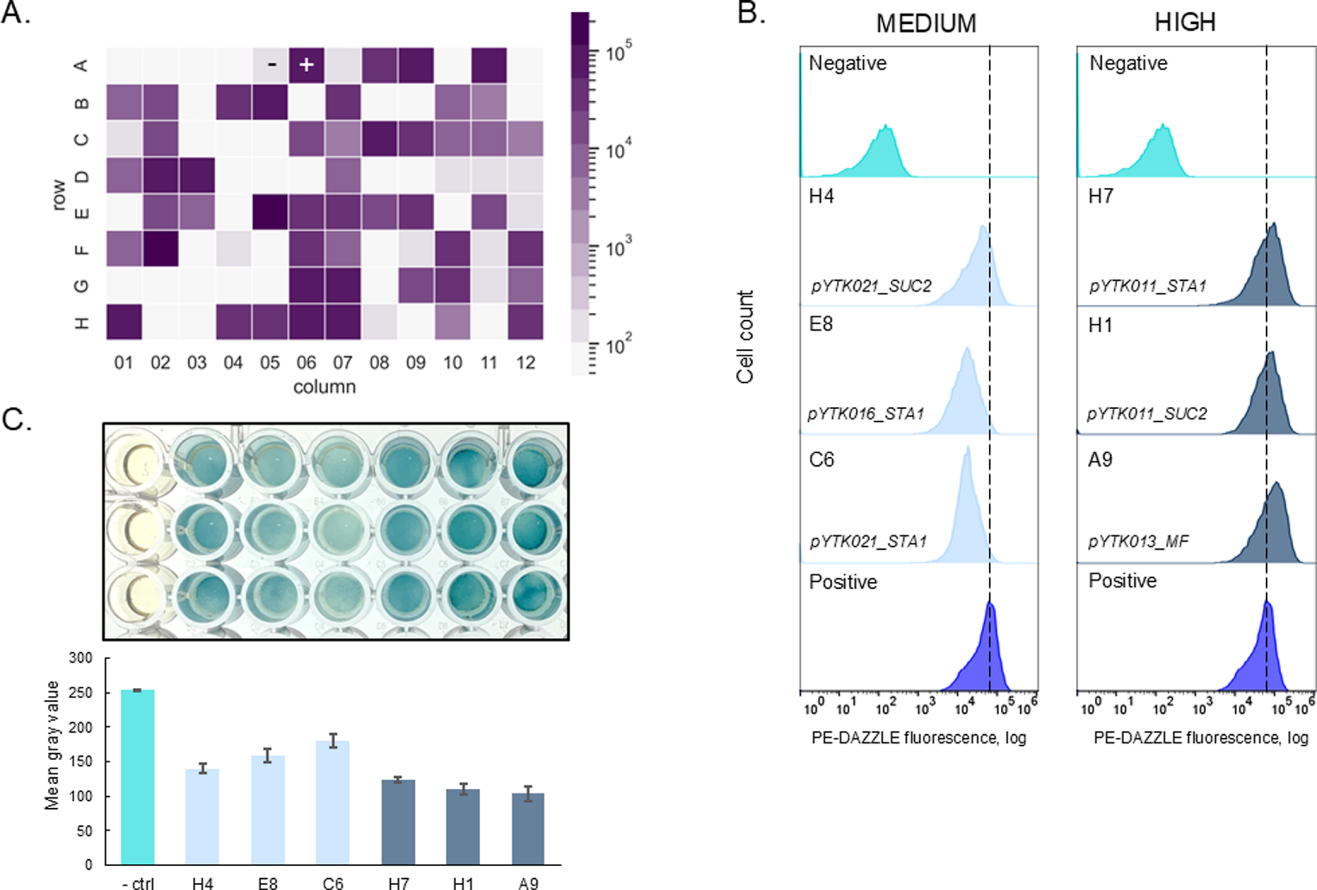
Single colony analysis of Mel1 surface display and secretion. (A) Heatmap of median PE fluorescence intensity of single colonies with Mel1 displayed. A single colony was picked and grown in a 96 well plate. Wells A1-A4 are empty, well A5 is the negative control (pYTK010 MF-3xFLAG-ELP1-CBM without staining) and well A6 is the positive control (pYTK010 MF-3xFLAG-ELP1-CBM stained). (B) PE fluorescence of selected strains with Mel1 surface display with medium and high fluorescence intensities: wells H4, E8, C6, H7, H1, and A9. Combinations of a promoter and a signal peptide for these strains were identified by Sanger sequencing. The highest levels of surface display were in wells E5 and F2, but sequencing showed that these strains do not have integration of the full-length genetic construct. Wells H1, B5, and D3 had an identical promoter and signal peptide combinations. (C) Alpha-galactosidase enzymatic activity assay. Supernatant of overnight cultures from 3 independent transformants was mixed with citric buffer and X-alpha-Gal. Images were made after 6 h of the reaction. The color intensity was analyzed with ImageJ as a gray value of a region of interest within each well. Negative control is the supernatant from BY4741. *n* = 3, error bars = standard deviation.

The identified promoters, signal peptides, Mel1, CBM, and HA-tTDH1, were cloned into an LEU2 integration vector without the surface display unit and transformed into the BY4741 wild-type strain. Yeast strains were cultured in rich media to a consistent OD_600_, and the supernatant was collected for analysis. Enzymatic activity was assessed using chromogenic substrate X-alpha-gal in citric buffer. The results corroborated the flow cytometry data: the negative control (BY4741 supernatant) showed no color development and the highest gray value. Strains corresponding to the medium surface display levels demonstrated a lighter coloration and lower enzyme secretion compared to those of high display and secretion strains. Remarkably, the enzymatic assay results precisely mirrored the flow cytometry findings: higher fluorescence levels directly corresponded to increased Mel1 secretion levels. This consistency validates the potential of our single colony analysis approach. The method’s versatility extends beyond enzymatically detectable proteins. For proteins lacking direct enzymatic assays, flow cytometry can initially identify the most promising candidates. Western blot analysis can then confirm the protein presence in the media, focusing resources on the most promising strains.

## Discussion

*S. cerevisiae* stands as a premier chassis for recombinant protein production, playing a critical role in synthesizing vital biopharmaceuticals, such as insulin and human serum albumin.^1–4^ The absence of a universal promoter-signal peptide combination for protein production in yeast necessitates unique optimization for each novel biopharmaceutical protein. There is a growing necessity for rapid high-throughput methods of protein secretion detection. Previous work from others demonstrated high-throughput methods for detecting secreted enzymes via 96 well plate assays^14^ or by employing microfluidics.^46^ Biosensor-based methods provide a solution for screening the secretion of nonenzyme proteins, but require single cell analysis.^15–17^ Our research presents a streamlined method for identifying optimal secretion strategies of enzymes and proteins without enzymatic activities, validated through both a high-throughput pooled library and single colony analysis approaches.

We successfully demonstrated the reliability of surface display as a secretion readout across four distinct synthetic proteins, including two enzymes and two structural proteins. The genetic design we propose, rooted in the widely used yeast MoClo toolkit,^41^ requires minimal modification to that cloning system, primarily just designing the protein of interest with adapted overhangs (Figure 1B,C). Our approach offers substantial flexibility, inviting the synthetic biology and biotechnology community to expand from this work, for example, by including further compatible promoters and signal peptides for specific research needs.

Previous studies have validated yeast surface display as an effective analytical platform for evaluating individual signal peptide-anchor protein combinations via flow cytometry, underscoring the critical principle that secretion optimization requires tailored approaches rather than universal solutions.^30^ Our method’s key strength of our method lies in its ability to generate combinatorial yeast libraries rapidly through a simple one-pot DNA assembly process, integrating various promoters and signal peptides with the protein of interest. Generation of yeast libraries followed by FACS allows fast strain characterization, while nanopore sequencing provides comprehensive genotype analysis of strains with different levels of fluorescence. The method also leverages genome integration for reliable single-copy expressions. The resulting yeast strains can be cultured without requiring selection, making them immediately applicable in both mono- and coculture environments.

The screening methodology demonstrated remarkable adaptability. Our high-throughput approach enables FACS-based library sorting into populations with distinct fluorescence intensities with promoter-signal peptide combinations efficiently characterized through a single nanopore sequencing run of barcoded amplicons. This strategy generates extensive experimental data, facilitating the identification of strains with varied secretion levels. For researchers seeking a more focused approach, our single colony analysis method provides an alternative. Utilizing flow cytometry and Sanger sequencing with nonbarcoded oligos—as demonstrated with alpha-galactosidase Mel1—offers a more targeted strain selection strategy.

An unexpected observation during single colony analysis revealed interesting nuances in the surface display. In rare instances, high fluorescence levels can result from the display of only a partial protein, potentially misleading strain classification. Such discrepancies can be readily identified through nanopore sequencing size-based read analysis or detected via colony PCR amplicon size variations. Intriguingly, these “false-high” strains with truncated displayed proteins unveiled an additional insight. Despite employing strong constitutive promoters for both AGA1 and coding sequence-AGA2 gene expression, the surface display capacity remains unsaturated for proteins of around 370 amino acids in length. This observation opens an exciting avenue for future research, particularly in screening and secreting smaller peptides.

Heterologous proteins can impose significant cellular stress that compromises both the viability and secretion efficiency. When surface display signals remain undetectable, inducible promoter systems offer a strategic alternative by decoupling growth and production phases, thereby minimizing the cytotoxic effects during cell propagation. The YTK provides an extensive collection of characterized inducible promoters,^41,47,48^ which can be incorporated into our toolkit.

Our research demonstrates that different promoters and signal peptides yield varied levels of surface display and secretion for beta-lactamase, ELP, and alpha-galactosidase Mel1 when fused to CBM. Notably, the optimal secreting strains for each of the four proteins required unique combinations of promoters and signal peptide. These findings underscore the protein-specific nature of successful secretion strategies. Our observations also reveal that secretion levels can vary significantly between different signal peptides used with the same promoter. This critical finding highlights that a strong promoter and high transcript production alone are insufficient to guarantee successful protein secretion. Each protein appears to demand a tailored approach to achieve the optimal secretion efficiency.

The method developed in this study offers two valuable approaches for research groups seeking to maximize protein yields. The high throughput method presents an efficient strategy for screening libraries of promoters and existing signal peptides and could also be used for identifying and quickly validating novel promoters and signal peptides. The data generated by the high throughput approach can also aid advanced computational techniques. The comprehensive data sets from it could serve as a valuable resource for machine learning applications, potentially enabling the engineering of synthetic signal peptides with enhanced secretion capabilities. At the same time, the low throughput version of the method is rapid and eliminates the need for complex data analysis, allowing one to focus resources on the best candidates.

## Materials and Methods

### Bacteria and Yeast Culture Conditions

*E. coli* NEB turbo was used for routine cloning, and NEB 10-beta (NEB, C3019) were used for the generation of libraries. Bacteria were grown in LB liquid (10 g/L Tryptone, 5 g/L Yeast Extract, 5 g/L NaCl) with aeration or on LB 2% agar at 37 °C. Chloramphenicol (34 μg/mL) or kanamycin (50 μg/mL) were added when appropriate. S. cerevisiae was grown in Yeast/Peptone/2% Dextrose at 30 °C with aeration to provide optimal conditions for yeast secreting high levels of proteins or on SC-LEU agar plates (1.4 g/l yeast synthetic dropout medium supplements, 6.8 g/l yeast nitrogen base without amino acids, and 20 g/l glucose and supplemented with tryptophan 50 mg/L, histidine 50 mg/L, and uracil 2 g/l) to select for successful transformants. The strains used in the study are listed in Table S1.

### Construction of Plasmids and Yeast Libraries

Plasmids used in the study are listed in Table S2. All molecular cloning steps were first performed in silico by using Benchling. The MoClo-YTK plasmid kit was a gift from John Dueber (Addgene kit #1000000061). Elastin-like polypeptide sequences were synthesized and cloned into pMA-RQ by Invitrogen. Oligos and (GS)_8_-Aga2-HA-tTDH1, (GS)_8_-HA-tTDH1, AMY1_SP_-3xFLAG, MFalpha_SP_-3xFLAG, STA1_SP_-3xFLAG, and SUC2_SP_-3xFLAG gBlocks were synthesized by IDT. Signal peptides and (GS)_8_-HA-tTDH1 were synthesized with BsmBI overhangs and cloned into pYTK001 (NEB, R0739). The resulting plasmids were confirmed by Sanger sequencing. (GS)_8_-Aga2-HA-tTDH1 fusion was synthesized as one part and cloned into LEU2 integration plasmid pWS064 via Gibson assembly according to the NEB protocol. The resulting destination plasmid pAK009 contained modified 4b part (GS)_8_-Aga2-HA-tTDH1, LEU2 homology arms, LEU2 gene, and *E. coli* GFP flanked by BsaI restriction sites to facilitate Golden Gate assembly of libraries. The presence of GFP dropout simplifies the selection of successful assemblies. The release of GFP generates an overhang compatible with the YTK genetic parts. The sequence of the resulting plasmid was confirmed by full plasmid sequencing (FullCircle). Parts 3a and 3b were designed to be compatible with YTK, while part 4a has a different overhang at 3′ end (modified from the original YTK to preserve (GS)_8_). All plasmids were designed to be assembled via Golden Gate reactions with BsaI restriction enzymes. Cellulose binding module (CBMcex) and Mel1 were amplified using Q5 polymerase (NEB, M0491) according to manufactures’ protocol from pCG05 and pCG21 ^44^ and cloned into pYTK001 as modified part 4a and 3a, respectively. Plasmid libraries were generated by the combinatorial assembly of promoters, signal peptides fused to FLAG tags and beta-lactamase, ELP2, ELP4, ELP6, ELP8, ELP10, and Mel1 coding sequences (3b parts), and CBMcex coding sequences (modified 4a part) into pAK009. The assembly was performed by Golden Gate reaction with BsaI-HF v2 (NEB, R3733) and T4 DNA ligase (NEB, M0202) according to the manufacturer’s protocol. Concentrations of all plasmids were measured with a broad range Quibit assay kit (ThermoFisher, Q32850) and normalized to 50 fmol before assembly into the backbone. A 1:2 ratio of backbone/ins was used. Assembly mixes were transformed into NEB 10-beta competent *E. coli* cells (NEB, C3019) according to the manufacturer’s protocol. Cells were plated onto LB + Kanamycin. White colonies for each assembly were collected as a pool for plasmid purification with a QIAprep Spin Miniprep Kit (Qiagen, 27104). Generated plasmid libraries were linearized with a NotI-HF restriction enzyme (NEB, R3189) and transformed into yeast via standard Lithium Acetate transformation.

### Flow Cytometry Analysis and Fluorescence Activated Cell Sorting

All yeast colonies grown after transformation were pooled and inoculated into 2 mL of YPD. One milliliter of this inoculate was diluted to 3 mL in YPD and grown for 3 h at 30 °C with aeration; the rest was frozen as glycerol stocks. After 3 h 150 μL of yeast cultures were washed with PBS and then resuspended in 150 μL of PBS containing FITC anti-HA.11 Epitope Tag Antibody (BioLegend, 901507) or PE/Dazzle 594 anti-DYKDDDDK Tag Antibody (anti-FLAG, BioLegend, 637329) at the same concentration of 0.2 μg/mL and incubated for 1 h protected from light. PBS/antibodies mix was then removed, and after one wash in PBS cells were resuspended in fresh PBS. Flow cytometry analysis was performed on an Attune NxT Flow Cytometer (Thermofisher) using Blue Laser 1 and Yellow Laser 2. At least 10,000 cells were analyzed for each sample. The data was processed with FlowJo version 9 (FlowJo, LLC) with appropriate gates applied. For better quantitative understanding of the range of detectable secretion from the flow cytometry data, dilution of PE-Dazzle exposed to a positive control yeast can be used with flow cytometry to determine if epitope occupancy limits the detection maxima. The titration of the cell number can also be performed with 5-fold serial dilutions of exponentially growing cultures, with these incubated with PE-Dazzle at 0.2 μg/mL and analyzed by flow cytometry, as shown in Figure S4.

For FACS cultures were diluted to 10 million cells/ml in PBS containing 0.2 μg/mL of PE/Dazzle 594 anti-DYKDDDDK Tag Antibody (BioLegend, 637,329), after 3 h of recovery and incubated for 1 h at room temperature protected from light. Cells were then washed in PBS and subjected to cell sorting. The sorting was performed with a BD FACSAria III (BD Biosciences) at the South Kensington Flow Cytometry Facility (Imperial College London, UK) using a Yellow-Green 561 nm laser. Three pools: low, medium, and high, were collected for each gene BLA, ELP2, and ELP4 based on PE/Dazzle intensity relative to stained cells without integration of Aga2 (no surface display, negative control) and cells with pCCW12 MF_3xFlag-ELP1-CMBcex-HA_Aga2_tTHD1 integrated into the *LEU* locus (high levels of surface display, positive control). Sorted pools were grown overnight in YPD at 30 °C with aeration to achieve dense populations for DNA extraction.

### Yeast Genomic DNA Extraction, Amplicons Generation, and Oxford Nanopore Sequencing

Genomic DNA was extracted from both unsorted populations and sorted pools using a Puregene Cell Kit (8 × 10^8^) (Qiagen, 158043) according to the manufacturer’s protocol. Integrated cassettes were amplified using Q5 High-Fidelity DNA Polymerase (NEB, M0491L) for 25 cycles with 25 ng of template DNA. PCR was set up with barcoded primers (Table S3) to allow the discrimination of the pools during the sequencing. The binding region was the same for all of the forward and reverse primers. The barcode in forward primers was unique for each gene encoded in the expression cassette (i.e., BLA, ELP2, or ELP4). Reverse primers contained unique barcodes to distinguish amplicons from low, medium, and high pools. PCR amplicons were purified with AMPure XP beads (Beckman Coulter, Brea, USA, A63880) using the same volume as the PCR reaction mix. The concentration of PCR amplicons was determined with a Qubit dsDNA BR kit (Invitrogen, Waltham, USA). Up to four pools were mixed in an equimolar amount (250 fmol of each pool) per sequencing run. Subsequently, PCR amplicons were prepared for nanopore sequencing using a ligation kit (LSK114, Oxford Nanopore Technologies, Oxford, UK). After adapter ligation, beads were washed with Short Fragments Buffer (SFB). The library was loaded on a Flongle flow cell and sequenced on a MinION device. The data was collected with MinKnow software (23.07.2015) and base calling was performed with Dorado (7.0.2 + 7e7b7d0).

### Nanopore Sequencing Data Analysis

Sequencing data was analyzed according to the POLAR-seq workflow we have previously described.^49^ Briefly, sequencing reads were filtered based on the presence of barcodes incorporated by PCR using demultiplexing function of Porechop.^50^ Four unique forward barcodes for combinatorial library of each ORF (i.e., ELP2, ELP4, and BLA) and three reverse barcodes for three levels of PE-DAZZLE intensity allow one to sequence 12 pools on a single flow cell (Table S3). Filtering reads based on the presence of both forward and reverse barcodes ensures that only reads spanning full amplicons are included in the analysis and is further stringent by filtered for the reads with a length of at least 2 kb. The resulting subset was converted from fastq to fasta and each read was annotated using Liftoff^51^ supplied with sequences of all genetic parts used for cloning as the reference. Generated annotation files (gff) were loaded into python with gffpandas and analyzed as pandas data frame. Read orientation was unified using the CBM sequence as a marker for 3′ end. Unique genotypes were counted using pandas and plotted with the seaborn package.

### Beta-lactamase Activity Assay

The presence of beta-lactamase in the supernatant was analyzed by a Biotek Synergy HT Multi-Mode Microplate Reader (Marchall Scientific). Yeast cultures from 3 independent transformants were grown in YPD overnight at 30 °C with aeration. The cultures were diluted 100 times in YPD and grown for 3 h at 30 °C with aeration. After cultivation, the cells were pelleted and 50 μL of supernatant were mixed with 50 μL of nitrocefin solution (50 μg/mL) for the beta-lactamase activity assay. The reaction was run for 1 h, and optical density was measured at 490 nm. Data was normalized to the cell density measured at 600 nm. A standard curve for enzymatic activity was generated with serial dilutions of beta-lactamase (ProSpec, ENZ-351) using mixing 50 μL of beta-lactamase solution with 50 μL of nitrocefin solution (50 μg/mL).

### Protein Concentration and Western Blot

The secretion efficiency of ELPs was analyzed by SDS-PAGE and Western blot. Yeast cells were grown in YPD overnight at 30 °C with aeration. Secreted proteins were precipitated from the supernatant using trichloroacetic acid (TCA). 1000 μL of the supernatant was mixed with 10 μL of 10% Sodium Deoxycholate, then 200 μL of 50% TCA were added and the tube was vigorously mixed. Precipitated proteins were pelleted at 4 °C for 30 min at maximum speed. The pellet was then washed with ice cold acetone and spun down at 4 °C for 20 min at maximum speed. The pellet was dried and resuspended in 50 μL of PBS and mixed with SDS sample buffer. Fifteen microliters of protein sample was loaded onto 4–20% precast protein gel (Biorad, 4561096). Fifteen microliters of GFP-HA purified in-house at different concentrations were used as a reference for the concentration of secreted proteins. The proteins were transferred onto a nitrocellulose membrane via wet transfer. The membranes were blocked in 5% milk/TBS-tween and incubated overnight with primary antibodies: anti-FLAG M2, 1:3000 in 5% milk/TBS-tween (Sigma, F3165), and anti-HA 1:5000 in 5% milk/TBS-tween (Invitrogen, 26183). After washing, membranes were incubated with antimouse HRP secondary antibodies used at 1:20000 concentration in 5% milk/TBS-tween (Invitrogen, 31430). Membranes were developed with SuperSignal West Pico PLUS Chemiluminescent Substrate (Thermo Scientific, 34577) and imaged with an Invitrogen iBright CL750 Imaging System.

### Alpha-Galactosidase Activity Assay

Yeast strains secreting Mel1 into media were grown overnight in YPD at 30 °C with aeration to OD_600_ = 20. A stock solution of 40 mg/mL of X-α-Gal (Sigma-Aldrich, 16555) was prepared in DMSO. For the alpha-galactosidase activity assay, 100 μL of the supernatant from overnight cultures were mixed with 5 μL of 100 mM citrate buffer pH 4.5 (45.95 mM sodium citrate dihydrate, 54.05 mM citric acid), and 10 μL of X-α-Gal were added to the cell mix. Pictures were made after 6 h from the start of the reaction. The mean gray value was measured for each well using ImageJ.

## Supplementary Material



## Notes

Flow cytometry data, protein quantification, plasmids, and strains and oligos used in the study (PDF)
