# Supplementary material for: YTK Display-and-Secrete: Screening for Optimal Protein Secretion Elements in Saccharomyces cerevisiae
Source: ACS Synth Biol. 2025 Aug 11;14(9):3473–86. doi: 10.1021/acssynbio.5c00264 (PMC12455636; doi:10.1021/acssynbio.5c00264)
Supplement: Supplementary file 1 [file sb5c00264_si_001.pdf]

Supporting Information

YTK Display-and-Secrete: Screening for Optimal Protein Secretion Elements in *Saccharomyces cerevisiae*

Anastasiya Kishkevich[1,2], Klaudia Ciurkot[1,2] and Tom Ellis*[1,2].

1. Imperial Centre for Engineering Biology, Imperial College London, London SW7 2AZ, UK
2. Department of Bioengineering, Imperial College London, London SW7 2AZ, UK

* Corresponding author *t.ellis@imperial.ac.uk*

Table of Contents

Supplementary Figure 1. Flow cytometry analysis of surface display of different ELP-CBM fusions. 2

Supplementary Figure 2. Standard curve for beta-lactamase activity 3

Supplementary Figure 3. Western blot analysis of secreted ELP-CBM fusions in the supernatant with purified GFP-HA tag protein at different concentrations. 4

Supplementary Figure 4. Titration of cell number for PE-Dazzle staining 5

Supplementary Table 1. Plasmids used in the study. 6

Supplementary Table 2. Basic strains used in the study 9

Supplementary Table 3. Barcoded oligos used for amplification integrated genomic constructs from yeast libraries and Nanopore sequencing 9

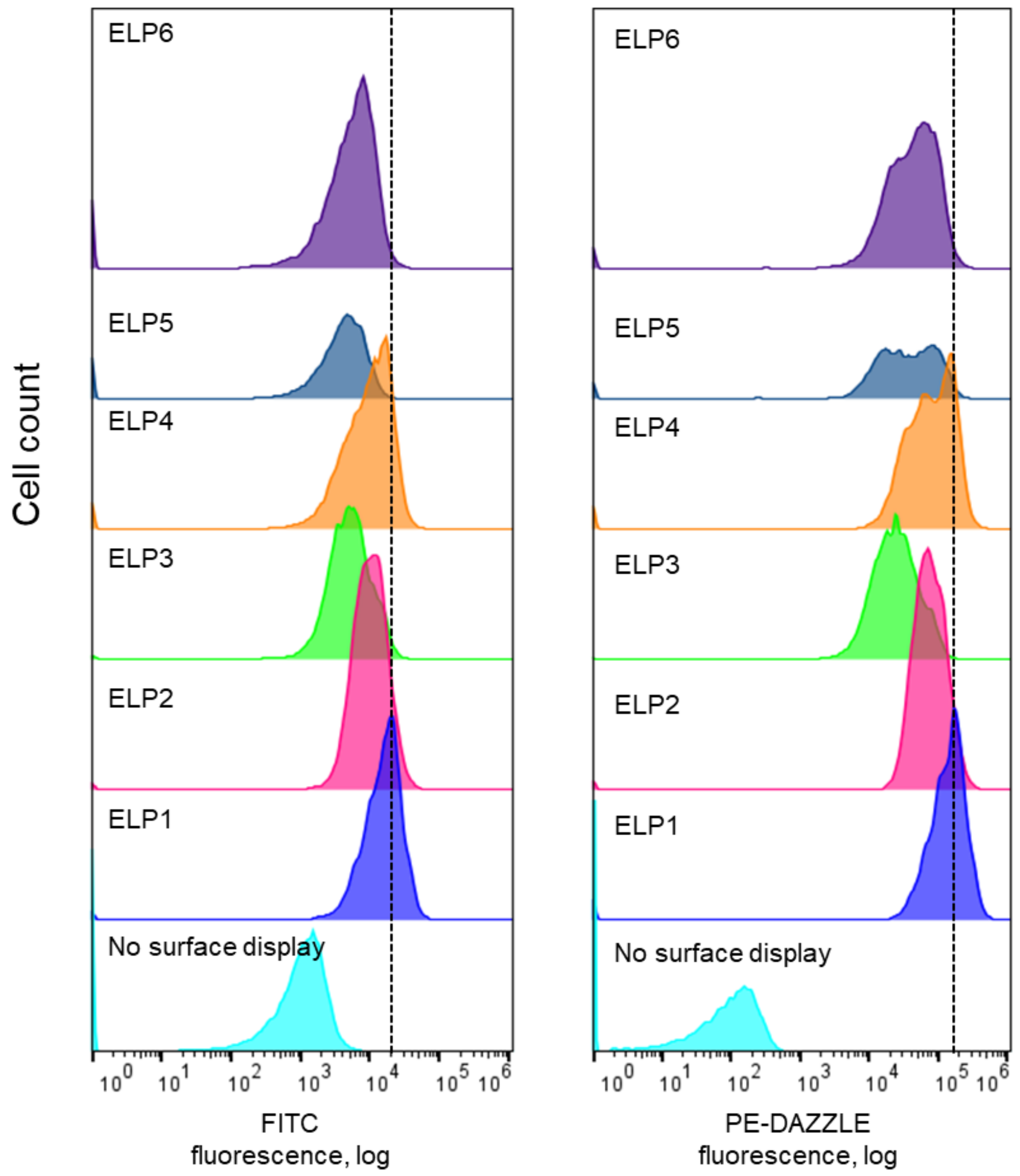

Supplementary Figure 1. Flow cytometry analysis of surface display of different ELP-CBM fusions. Cells were treated with FITC-anti-HA and PE-DAZZLE antibodies and analysed by Attune NxT Flow Cytometer. At least 10000 events were analysed for each sample. A dashed line indicates median fluorescence level for ELP1-CBM fusion.

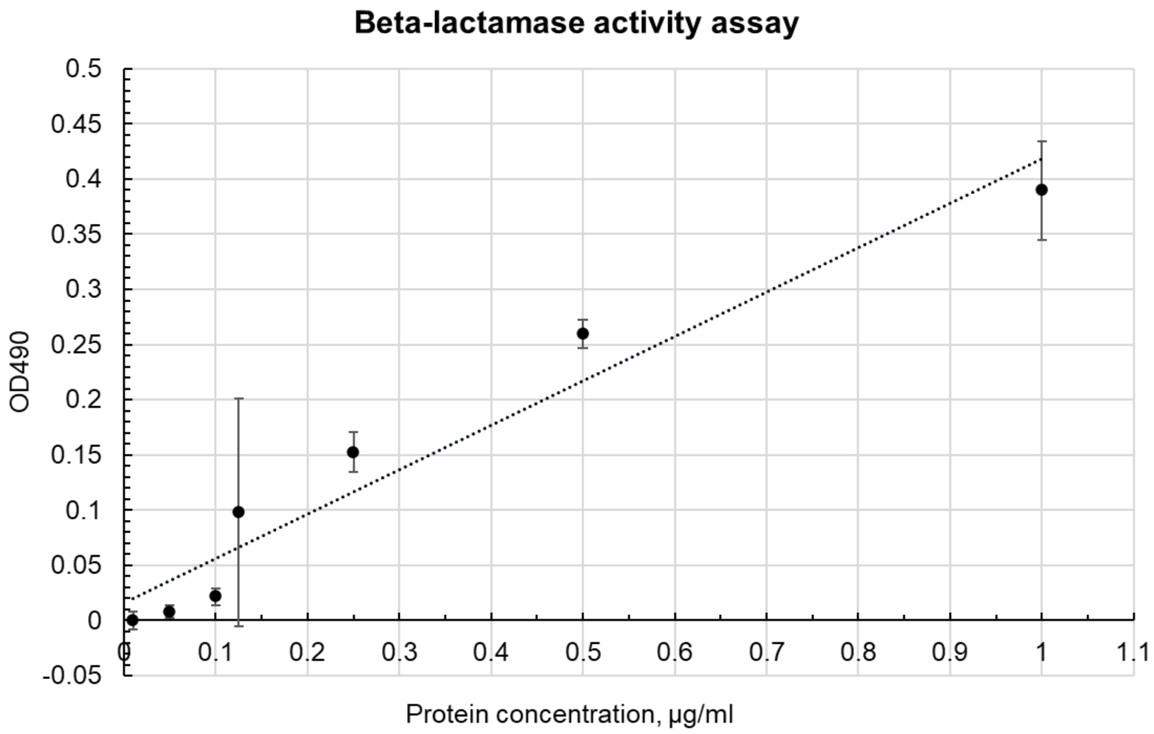

Supplementary Figure 2. Standard curve for beta-lactamase activity. Beta-lactamase enzymatic activity assay was performed with different concentrations of commercial enzyme mixed with equal volume of 50 µg/ml of nitrocefin solution. Data is presented as average of OD490 reads from at least two technical repeats.

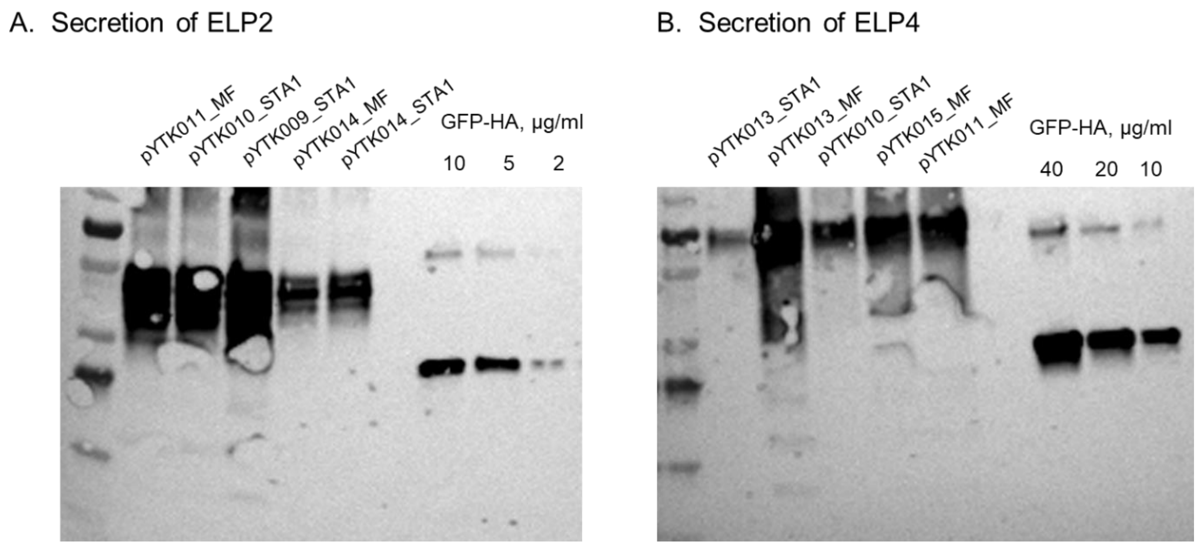

Supplementary Figure 3. Western blot analysis of secreted ELP-CBM fusions in the supernatant with purified GFP-HA tag protein at different concentrations. Supernatants were concentrated 20 times after TCA extraction, mixed with 6X sample buffer and 15 µl was loaded on the gel along with 15 µl of reference protein. Exposure time 45 s (A) and 10 s (B). Actual concentration of ELP-CBM in the supernatant are 20 times less than in loaded samples.

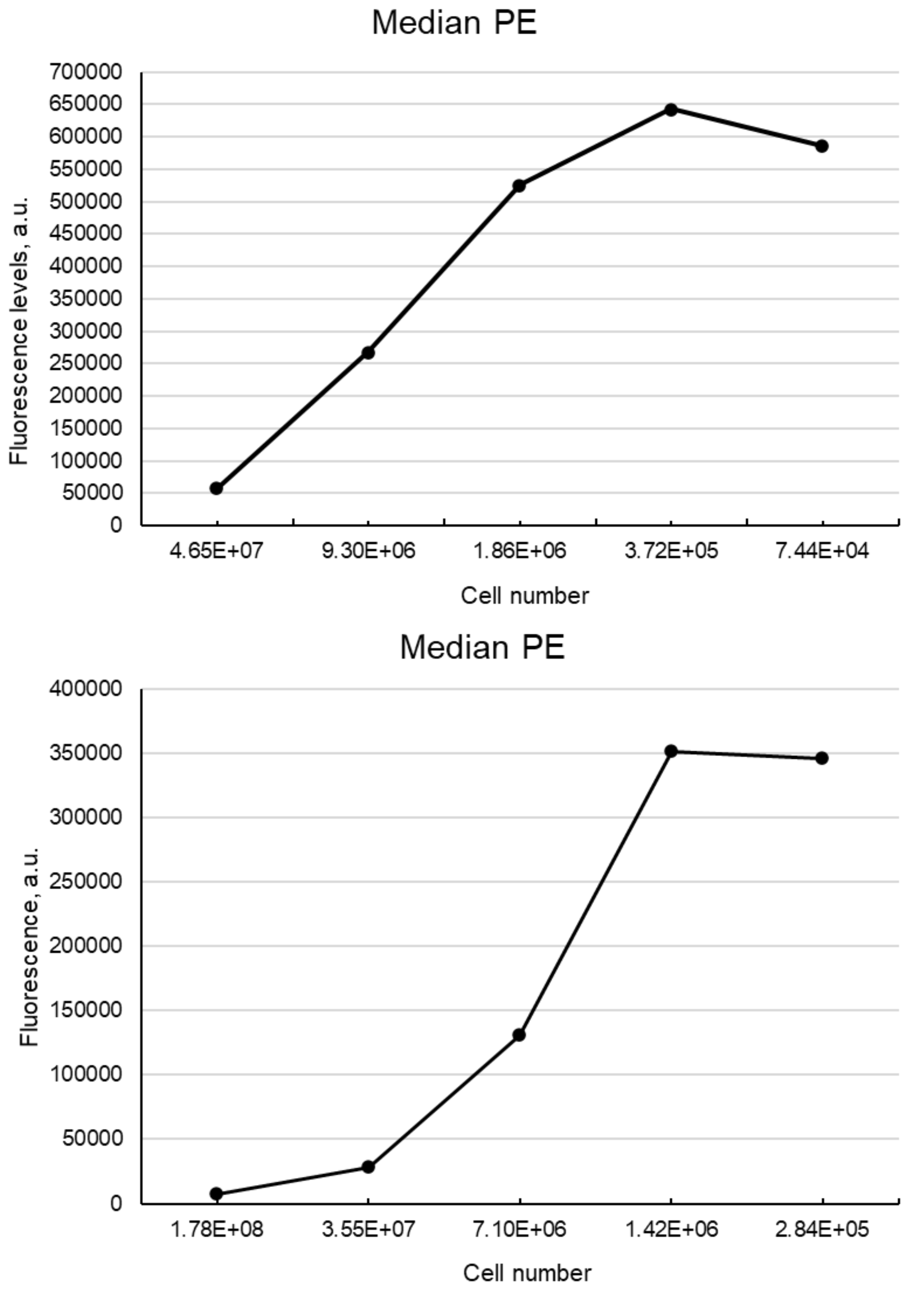

Supplementary Figure 4. Titration of cell number for PE-Dazzle staining. The high secreting strain was 5-fold serially diluted and incubated with the same concentration of PE-Dazzle antibodies according to the protocol described in the Methods section. Graphs represent 2 independent experiments.

Supplementary Table 1. Plasmids used in the study.

Plasmid	Construct details	Source
**pYTK001**	Entry level vector from YTK used to clone modular parts by Golden Gate assembly via BsmBI sites	Lee et al.
**pYTK008**	*E. coli* ConLS' in pYTK001	Lee et al.
**pYTK009**	TDH3 promoter in pYTK001, YTK part 2	Lee et al.
**pYTK010**	CCW12 promoter in pYTK001, YTK part 2	Lee et al.
**pYTK011**	PGK1 promoter in pYTK001, YTK part 2	Lee et al.
**pYTK012**	HHF2 promoter in pYTK001, YTK part 2	Lee et al.
**pYTK013**	TEF1 promoter in pYTK001, YTK part 2	Lee et al.
**pYTK014**	TEF2 promoter in pYTK001, YTK part 2	Lee et al.
**pYTK015**	HHF1 promoter in pYTK001, YTK part 2	Lee et al.
**pYTK016**	HTB2 promoter in pYTK001, YTK part 2	Lee et al.
**pYTK017**	RPL18B promoter in pYTK001, YTK part 2	Lee et al.
**pYTK018**	ALD6 promoter in pYTK001, YTK part 2	Lee et al.
**pYTK019**	PAB1 promoter in pYTK001, YTK part 2	Lee et al.
**pYTK020**	RET2 promoter in pYTK001, YTK part 2	Lee et al.
**pYTK021**	RNR1 promoter in pYTK001, YTK part 2	Lee et al.
**pYTK022**	SAC6 promoter in pYTK001, YTK part 2	Lee et al.
**pYTK024**	POP6 promoter in pYTK001, YTK part 2	Lee et al.
**pYTK025**	RAD27 promoter in pYTK001, YTK part 2	Lee et al.
**pYTK026**	PSP2 promoter in pYTK001, YTK part 2	Lee et al.
**pYTK027**	REV1 promoter in pYTK001, YTK part 2	Lee et al.
**pYTK028**	MFA1 promoter in pYTK001, YTK part 2	Lee et al.
**pYTK029**	MFα2 promoter in pYTK001, YTK part 2	Lee et al.
**pYTK055**	ENO1 terminator in pYTK001, YTK part 4	Lee et al.
**pYTK075**	*S. cerevisiae* LEU2 gene in pYTK001	Lee et al.
**pYTK087**	*S. cerevisiae* LEU2 3' Homology in pYTK001	Lee et al.
**pYTK090**	*E. coli* KanR-ColE1 in pYTK001	Lee et al.
**pYTK093**	*S. cerevisiae* LEU2 5' Homology region in pYTK001	Lee et al.
**pWS2364**	One repeats of synthetic Elastin Like Polypeptide (ELP1) in pYTK001, YTK part 3b	Ellis group collection
**pWS2365**	Two repeats of synthetic Elastin Like Polypeptide (ELP2) in pYTK001, YTK part 3b	Ellis group collection

**pWS2367**	Four repeats of synthetic Elastin Like Polypeptide (ELP4) in pYTK001, YTK part 3b	Ellis group collection
**pWS2369**	Six repeats of synthetic Elastin Like Polypeptide (ELP6) in pYTK001, YTK part 3b	Ellis group collection
**pWS2371**	Eight repeats of synthetic Elastin Like Polypeptide (ELP8) in pYTK001, YTK part 3b	Ellis group collection
**pWS2373**	Ten repeats of synthetic Elastin Like Polypeptide (ELP10) in pYTK001, YTK part 3b	Ellis group collection
**pWS032**	*E. coli* TEM1 beta-lactamase gene BLA in pYTK001, protein lacking signal peptide (UniProt: Q6SJ61), YTK part 3b	Gilbert et al.
**pPPK046**	*S. cerevisiae* alpha-galactosidase 1 MEL11 in pYTK001, protein lacking signal peptide (UniProt: P04824), YTK part 3b	Gilbert et al.
**pKK013**	*S. cerevisiae* AGA1 coding sequence (YNR044W) in pYTK001, full length protein, YTK part 3	Ellis group collection
**pAK005**	*Cellulomonas fimi* exoglucanase/xylanase cellulose binding module in pYTK001 (UniProt: P07986), YTK modified part 4a	This study
**pAK070**	*Aspergillus spp* alpha-amylase signal peptide fused to three *DYKDDDDK* repeats (3xFLAG) in pYTK001, YTK part 3a	This study
**pAK071**	*S. cerevisiae* mating pheromone MFA1 (YDR461W) signal peptide and pro-leader sequence fused to three *DYKDDDDK* repeats (3xFLAG) in pYTK001, YTK part 3a	This study
**pAK072**	*S. cerevisiae* glucoamylase STA1 (YSC0033) signal peptide fused to three *DYKDDDDK* repeats (3xFLAG) in pYTK001, YTK part 3a	This study
**pAK073**	*S. cerevisiae* invertase SUC2 (YIL162W) signal peptide fused to three *DYKDDDDK* repeats (3xFLAG) in pYTK001, YTK part 3a	This study
**pAK011**	HA epitope tag and THD1 terminator in pYTK001, YTK modified part 4b	This study
**pYTK096**	Pre-assembled YTK plasmid used to assemble all YTK parts together by Golden Gate assembly; contains KanR-ColE1 and GFP drop out for cloning in *E. coli* and URA3 3' and 5' homology arms and URA3 gene for integration into yeast genome.	Lee et al.

**pWS064**	Pre-assembled plasmid used to assemble all parts together by Golden Gate assembly; contains KanR-ColE1 and GFP drop out for cloning in *E. coli* and LEU 3' and 5' homology arms and LEU2 gene for integration into yeast genome. Generated by Golden Gate assembly via BsaI sites in the following order pYTK008, pYTK047, pYTK087, pYTK073, pYTK093, pYTK090 and pYTK075	Ellis group collection
**pAK009**	Surface display plasmid carrying *S. cerevisiae* AGA2 (YGL032C) gene without start codon and signal peptide fused to HA epitope tag and THD1 terminator; contains KanR-ColE1 and GFP drop out for cloning in *E. coli* and LEU2 3' and 5' homology arms and LEU2 gene for integration into yeast genome. Generated by Gibson assembly of amplicons of pWS064 and AGA2-HA tag-tTDH1 gBlock (synthetised by IDT)	This study
**pAK056**	Integration plasmid for *S. cerevisiae* containing CCW12 promoter, AGA1 and ENO1 terminator in pYTK096; generated by Golden Gate assembly of pYTK010, pKK013, pYTK055 and pYTK096	This study

Supplementary Table 2. Basic strains used in the study.

Strain	Description	Source
**BY4741**	*MATa his3Δ1 leu2Δ0 met15Δ0 ura3Δ0*	Dharmacon yeast collection
**yWO3485**	*MATa his3Δ1 leu2Δ0 met15Δ0 ura3Δ0 aga1Δaga2Δ*	Ellis group collection
**yAK03 (AGA1+)**	*MATa his3Δ1 leu2Δ0 met15Δ0 ura3Δ0 aga1Δaga2Δ pCCW12-AGA1-tENO1* integrated into *URA* locus for constitutive expression.	This study
**yAK04 (ELP1 positive control)**	*MATa his3Δ1 leu2Δ0 met15Δ0 ura3Δ0 aga1Δaga2Δ pCCW12-AGA1-tENO1* integrated into *URA* locus for constitutive expression, *pCCW12-MFαsp-3xFLAG-ELP1-CBM-AGA2-HA-tTDH1* integrated into *LEU* locus for constitutive expression	This study

Supplementary Table 3. Barcoded oligos used for amplification integrated genomic constructs from yeast libraries and Nanopore sequencing

Oligo	Definition	Sequence
oAK179	RV_barcode_Low	***catccaacactctacgccctcttca***CAGACCCAAAAACATACTGTGT
oAK180	RV_barcode_Medium	***cgtgttcgtttactgttaattggtg***CAGACCCAAAAACATACTGTGT
oAK181	RV_barcode_High	***gcacataagcaatatcgtagtccgt***CAGACCCAAAAACATACTGTGT
oAK182	FW_barcode_ELP2	***atgagtgtcagcgagtgtaactcga***CTGAATTCGCATCTAGACTGAT
oAK183	FW_barcode_ELP4	***ttgtactaatcggcttcaacgtgcc***CTGAATTCGCATCTAGACTGAT
oAK187	FW_barcode_beta-lactamase	***tagtttcgaaccacggttactaatc***CTGAATTCGCATCTAGACTGAT
